# Fetal Ovarian Cyst Associated With Disorders of Sex Development: A Case Report

**DOI:** 10.7759/cureus.101022

**Published:** 2026-01-07

**Authors:** Ana Sofia Rosado-Guzmán, Zaira Vanessa Escobedo-Enríquez, Abril Adriana Arellano-Llamas, Omar Rodolfo Sánchez Balpuesta, Cristo Neftaly Pérez-Lemus

**Affiliations:** 1 Obstetrics and Gynecology, Hospital de Gineco Obstetricia No. 3 "La Raza" Medicina Materno Fetal, Instituto Mexicano del Seguro Social, Mexico City, MEX; 2 Pediatrics/Endocrinology, Hospital de Gineco Obstetricia No. 3 "La Raza" Instituto Mexicano del Seguro Social, Mexico City, MEX; 3 Pediatrics/Neonatalogy, Hospital de Gineco Obstetricia No. 3 "La Raza" Neonatología, Instituto Mexicano del Seguro Social, Mexico City, MEX; 4 Pediatric Surgery/New Born Surgery, Hospital General "La Raza" Cirugía Pediátrica, Instituto Mexicano del Seguro Social, Mexico City, MEX

**Keywords:** ambiguous genitalia, congenital adrenal hyperplaisa, ovarian cyst, prenatal diagnosis, ultrasound fetal

## Abstract

Ovarian cyst is a frequent abdominal tumor in female fetuses (1/2,600 live births), but its prenatal diagnosis can be challenging in the context of ambiguous genitalia. The most common cause of genital virilization in female fetuses is congenital adrenal hyperplasia (CAH), which results from the overproduction of androgen hormones. These hormones may influence the fetal ovary in a way comparable to polycystic ovarian syndrome (PCOS) in adults, leading to the development of an ovarian cyst that may regress. We present the case of a fetus prenatally diagnosed with a large ovarian cyst and ambiguous genitalia. After birth, a diagnosis of salt-wasting CAH was confirmed. A left salpingo-oophorectomy was performed, and histopathological examination revealed a simple follicular cyst.

## Introduction

Ovarian cysts are the most common abdominal masses identified in female fetuses and newborns, occurring in approximately 1 in 2,600 live births. They are typically detected during the third trimester, and the most widely accepted mechanism involves overstimulation of the fetal ovaries by maternal and placental gonadotropins and estrogens. Additional maternal factors associated with their development include diabetes, preeclampsia, Rh alloimmunization [1], and androgen exposure [2]. Congenital adrenal hyperplasia (CAH) is the leading cause of virilized external genitalia in newborns with a 46,XX karyotype. It is a potentially life-threatening endocrine disorder if diagnosis or treatment is delayed, which is why CAH is part of universal newborn screening programs in Mexico during the first days of life. The condition arises from impaired enzyme activity in steroid biosynthesis, resulting in cortisol deficiency and, in some cases, aldosterone deficiency, along with the accumulation of adrenal androgen precursors [2].

Excess androgen exposure during fetal life not only disrupts normal genital differentiation but may also impact ovarian development. Androgens can increase follicular sensitivity to gonadotropins, particularly follicle-stimulating hormone (FSH), thereby promoting follicular growth. In this context, sustained hyperandrogenism combined with gonadotropin overstimulation may lead to follicular enlargement and the subsequent formation of fetal ovarian cysts in affected fetuses [3,4]. This case is unique because of the prenatal detection of a large ovarian cyst in a fetus with CAH, a rarely reported association. Reporting this case adds to the limited existing literature and highlights the importance of considering underlying endocrine disorders in the evaluation of fetal ovarian cysts.

## Case presentation

**Figure 1:**
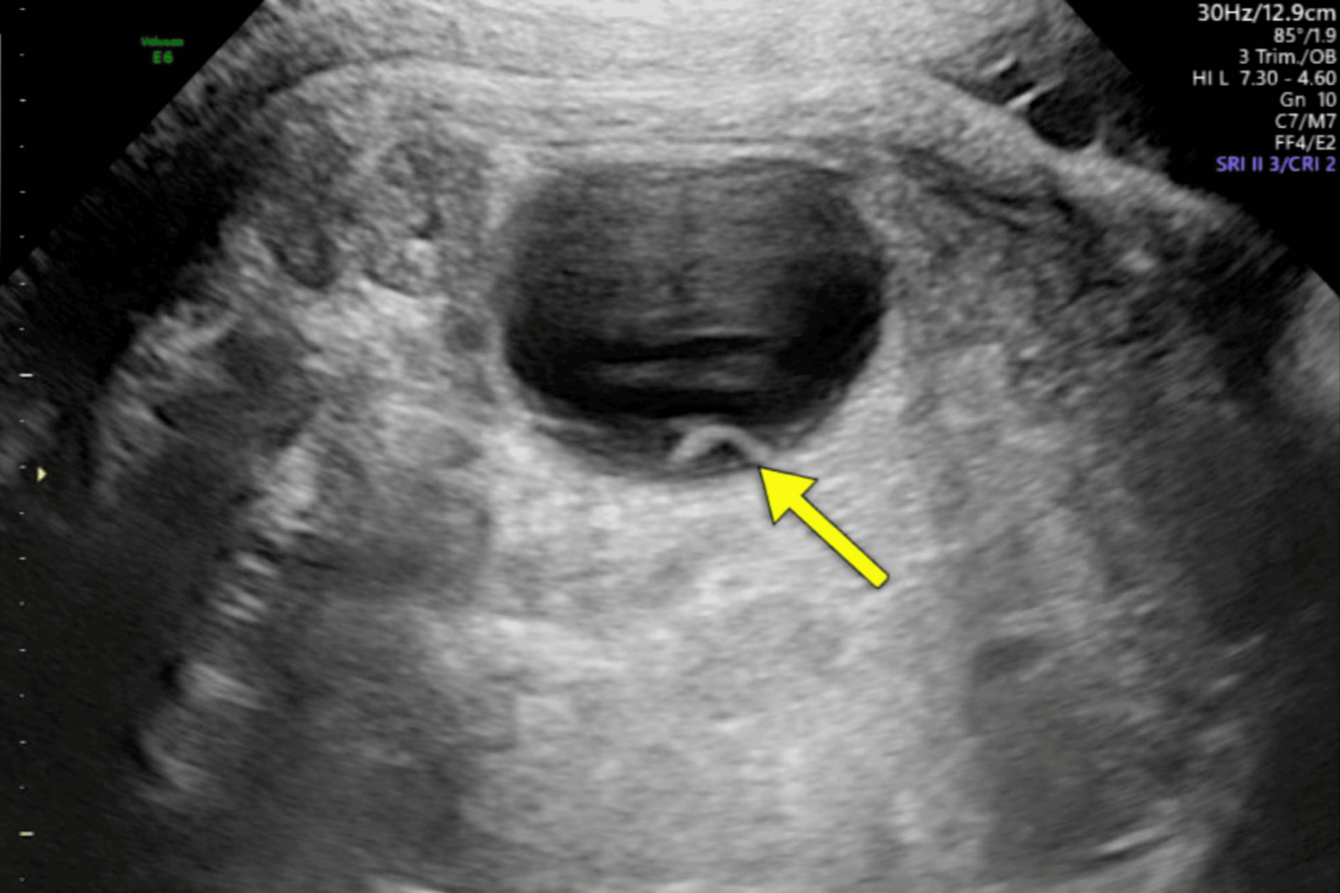
Fetal ovarian cyst Fetal echography at 39 weeks demonstrating a round anechoic cystic lesion (49.3 × 42.2 × 41.0 mm) with an internal “daughter cyst” (arrow), consistent with an ovarian cyst

We present the case of a 39-week fetus in whom ultrasound revealed a large left ovarian cyst, along with a smaller internal "daughter cyst", suggestive of ovarian origin (Figure 1). The external genitalia showed a phallic structure and prominent labioscrotal folds, raising suspicion of ambiguous genitalia. The Müllerian structures and gonads were not clearly visualized during the ultrasound.

**Figure 2:**
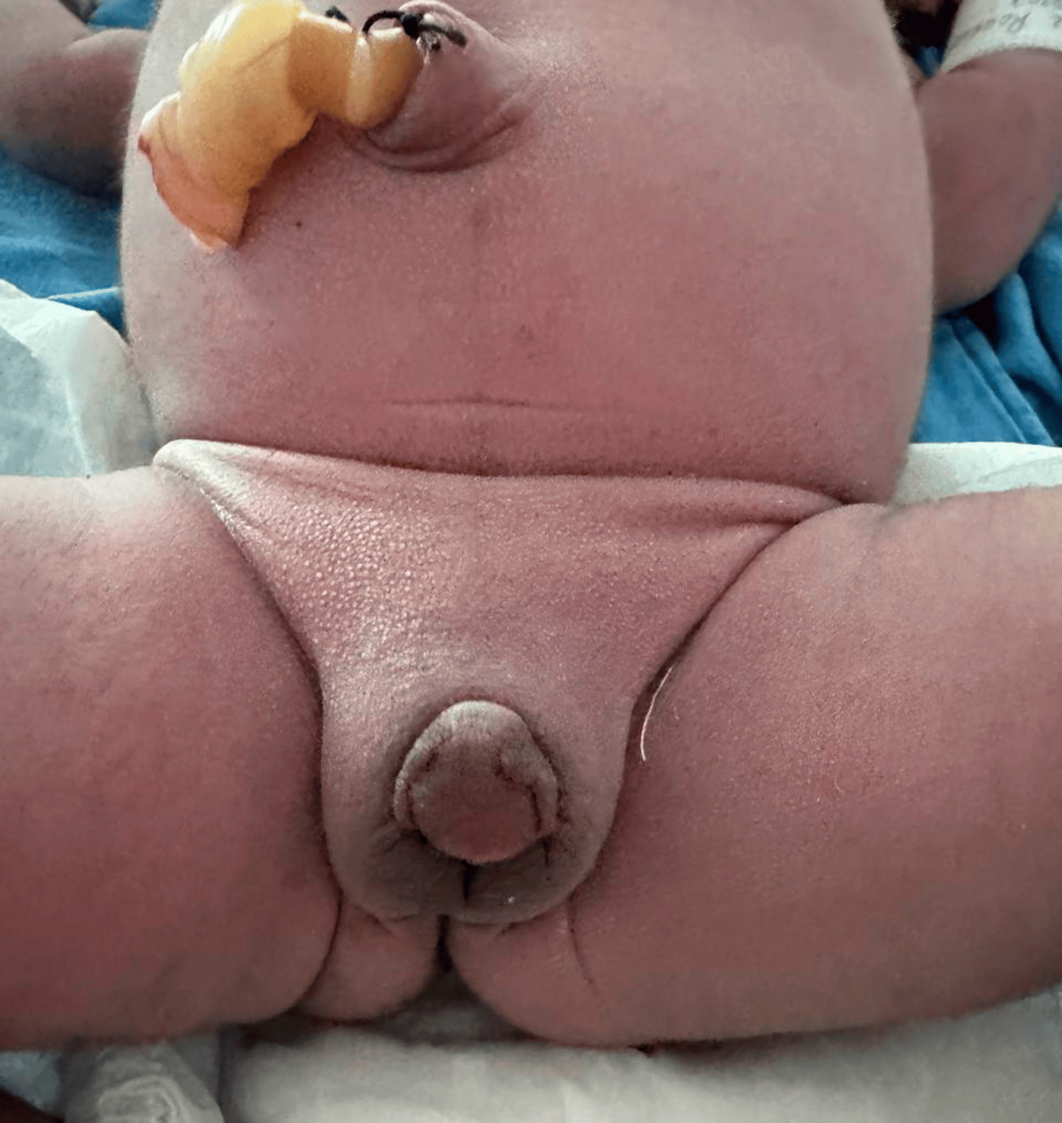
Ambiguous genitalia Genital examination showed a 2.5-cm phallus, and fused labioscrotal folds were noted, along with a solitary urogenital orifice positioned at the base of the phallus

The newborn was delivered by cesarean section due to the high risk of ovarian torsion, weighing 3600 g at birth and measuring 50 cm in length, with Apgar scores of 8/9 (at one and five minutes, respectively). Postnatal examination confirmed severe virilization, consisting of fused labioscrotal folds, a 2.5 cm phallus, a single urogenital orifice, hyperpigmented external genitalia, and absence of palpable gonads (Figure 2). The virilization was classified as Prader 5.

**Figure 3:**
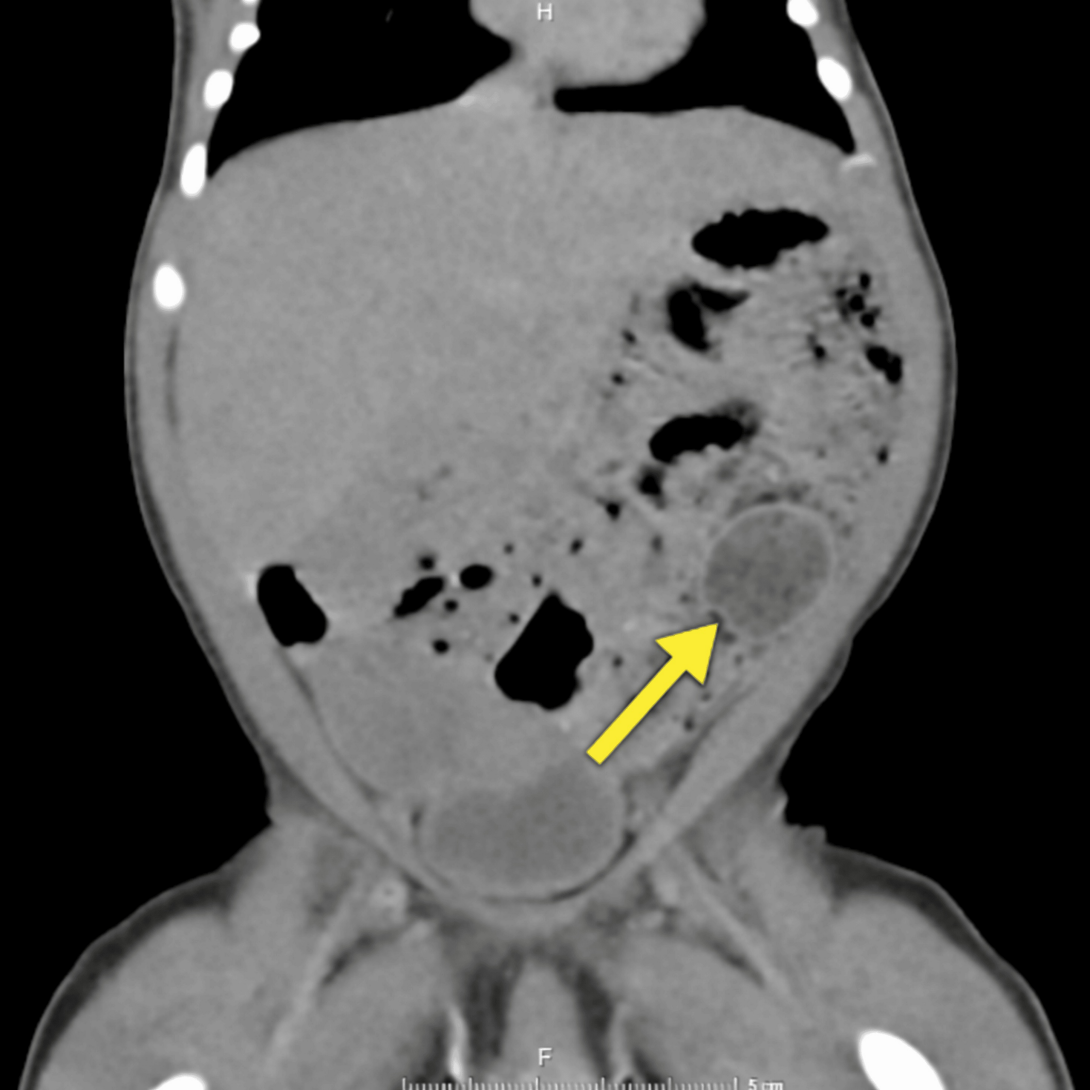
CT scan findings Multislice scan of the abdomen and pelvis demonstrating a well-defined mass in the left iliac fossa measuring 29.2 × 20.8 × 26.2 mm (arrow) CT: computed tomography

Abdominopelvic ultrasound revealed a large cystic structure occupying the pelvic-abdominal region, measuring 52 × 45 × 57 mm, of predominantly ovarian origin. Müllerian structures were visualized. Transverse abdominopelvic CT showed a well-defined lesion in the left iliac fossa, measuring 29.2 × 20.8 × 26.2 mm (Figure 3), along with bilateral adrenal gland thickening and a probable left ureterocele.

Voiding cystourethrography revealed findings consistent with a left ureterocele, high-pressure vesicoureteral reflux, and features suggestive of a neurogenic bladder. Laboratory evaluation demonstrated a marked increase in androgen precursors and electrolyte imbalances consistent with salt-wasting CAH (Table 1). Karyotyping confirmed a 46,XX karyotype.

**Table 1:** Laboratory findings Laboratory evaluation showed markedly elevated androgen precursors and electrolyte abnormalities consistent with salt-wasting congenital adrenal hyperplasia FSH: follicle-stimulating hormone; LH: luteinizing hormone

Parameter	Result	Reference range	Interpretation
17-hydroxyprogesterone	>30 nmol/L	0.07–0.77 nmol/L	Increased
Serum testosterone	7.50 ng/mL	0.2–0.64 ng/mL	Increased
Cortisol (8 AM)	1.96 µg/mL	10–20 µg/mL	Decreased
FSH	<0.30 mUI/mL	0.2–9.2 mUI/mL	Decreased
LH	<0.10 mUI/mL	0.02–7.0 mUI/mL	Decreased
Sodium	133 mmol/L	135–145 mmol/L	Decreased
Potassium	6.1 mmol/L	3.5–5.5 mmol/L	Increased

**Figure 4:**
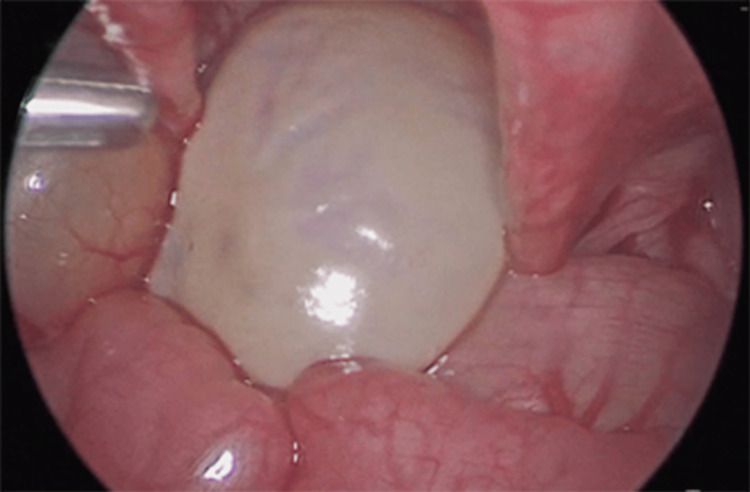
Laparoscopy findings Diagnostic and therapeutic laparoscopy found an ovarian cyst on the left ovary measuring 5 x 3 x 3 cm

Salt-wasting CAH was diagnosed and treated with a high dose of hydrocortisone (50 mg/m²). Given the size of the cyst and the risk of torsion, a therapeutic laparoscopy was performed, revealing a 5 x 3 x 3 cm cyst occupying the left ovary (Figure 4), typical Müllerian structures, and a urogenital sinus. A left salpingo-oophorectomy, ureteral reimplantation, and placement of a double-J stent were performed.

**Figure 5:**
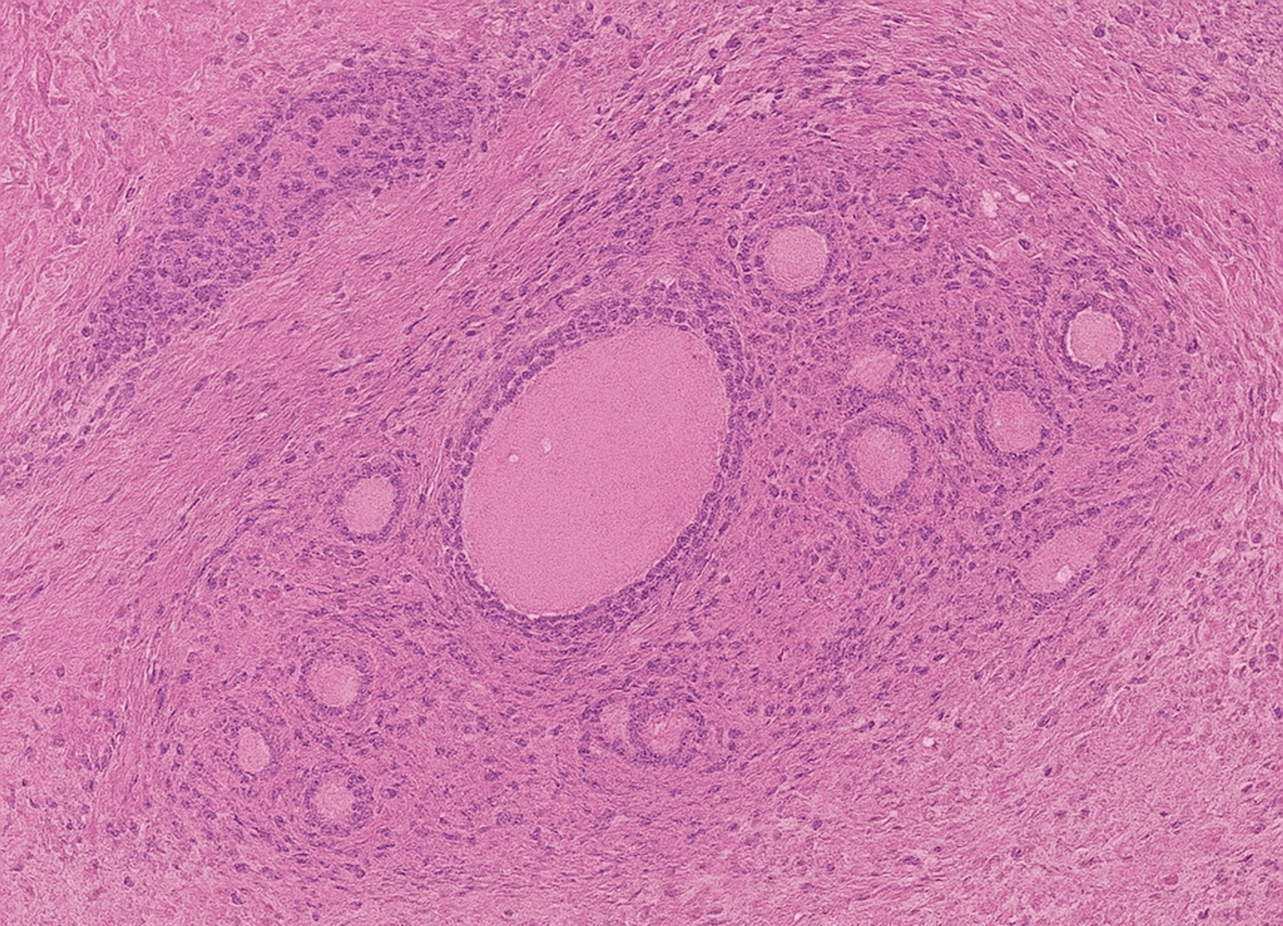
Pathological evaluation Low-magnification hematoxylin and eosin (H&E) microphotograph showing ovarian stroma with follicular structures compatible with a functional follicular cyst

Pathological evaluation, using hematoxylin and eosin staining along with inhibin immunohistochemistry, identified the lesion as a benign functional follicular cyst (Figure 5). The inhibin photomicrograph was not available for publication.

The baby developed a nosocomial urinary tract infection with fever and leukocytosis; urine culture revealed E. coli >100,000 CFU, which was successfully treated with meropenem.

## Discussion

Most fetal ovarian cysts are benign functional lesions that typically appear during the third trimester and are thought to arise from increased fetal exposure to maternal and placental hormones. Despite their benign nature, they can progress to more serious complications, such as torsion, compression of adjacent structures, rupture, intracystic hemorrhage, or even autoamputation, any of which may require surgical intervention [5]. These cysts occur in more than 80% of newborn girls and can reach up to 20 mm in size; most are unilateral on the left side (44%), followed by the right side (31%) and bilateral cases (25%) [6]. The "daughter cyst" sign describes a small, round, anechoic structure within a cyst and is pathognomonic of ovarian origin.

The differential diagnosis includes renal or urinary tract cysts (e.g., ureterocele, urinoma), urachal remnants, dilated bowel loops, meconium pseudocysts, enteric duplication cysts, lymphangiomas, choledochal cysts, and neuroblastomas in chemical forms [7]. Nussbaum et al. proposed a classification based on ultrasound and histopathological findings. Simple cysts are anechoic, thin-walled, and usually unilocular structures located on one side, while complex cysts have thicker walls, septa, solid components, or internal echoes [6]. An accurate differential diagnosis is essential to determine the organ of origin and to plan appropriate treatment [6,8].

Several authors have suggested that prenatal drainage of large fetal ovarian cysts, particularly those exceeding 4 cm, may help reduce the likelihood of complications. An exploratory review conducted by Bucuri et al. found that complex cysts are associated with roughly a one-third higher complication rate across 15 studies [9,10]. In a large series of 82 fetal ovarian cysts, most were managed expectantly. Despite conservative treatment, many developed intracystic hemorrhage, which exacerbates the poor ovarian prognosis associated with internal bleeding. It remains unclear whether prophylactic aspiration provides any benefit. In bilateral cases, delivery after lung maturity may allow for timely surgical intervention to preserve ovarian tissue [11]. Although surgery is often employed, it carries neonatal risks and does not always preserve ovarian function [1]. In our case, a unilateral salpingo-oophorectomy was necessary due to complete involvement of the ovary.

In classic CAH due to 21-hydroxylase deficiency, impaired cortisol synthesis leads to chronically elevated ACTH and excessive adrenal androgen production. Prenatal exposure to high levels of androgens such as testosterone and androstenedione during critical developmental periods results in virilization of the external genitalia in genetically defined females [12]. In addition to their effects on genital differentiation, hyperandrogenic states can also influence ovarian development. Experimental data suggest that androgens can increase follicular sensitivity to FSH by increasing the expression of FSH receptors, thereby promoting follicular growth and cyst formation, similar to the mechanisms observed in polycystic ovary syndrome (PCOS) in adults [13]. In addition, excess adrenal steroids can interfere with the normal regulation of gonadotropin secretion or exert direct effects on ovarian tissue, further contributing to the development of cysts [2,4].

These mechanisms may act synergistically in neonates with salt-wasting CAH, providing a plausible explanation for the appearance of ovarian cysts in this population, as observed in the present case. In this patient, the abdominal mass and the presence of a "daughter cyst" were characteristic of an ovarian cyst; however, the presence of ambiguous genitalia and the inability to visualize Müllerian structures on prenatal imaging made the diagnosis of CAH more challenging. The mother had no signs of virilization or exposure to androgenic medications. After birth, virilization at Prader stage 5, elevated androgen levels, electrolyte imbalances, low cortisol, elevated 17-OHP levels, and the visibility of Müllerian structures supported the diagnosis of salt-wasting 21-hydroxylase deficiency. Aldosterone and renin testing were not required. Given that fludrocortisone is difficult to obtain in Mexico, high-dose hydrocortisone was administered until discharge.

Newborn screening is essential for the early detection of CAH, allowing for the immediate initiation of corticosteroid treatment and reducing mortality. Children with CAH face risks related to both the disease and its treatment, such as linear growth retardation and pubertal disturbances, which require structured follow-up [3]. Previous case reports have described the association between CAH, particularly classic 21-hydroxylase deficiency, and the development of ovarian cysts in neonates and infants. Most reported cases presented with severe hyperandrogenic states and virilized external genitalia, supporting the role of excess adrenal androgens in ovarian follicular overstimulation. However, due to the limited number of reported cases, the true incidence and pathophysiology of this association remain incompletely understood [2,4].

The salt-wasting form carries a high risk of adrenal crisis and death. Although many cysts resolve, persistent stimulation of the hypothalamic-pituitary-ovarian axis can predispose to torsion or hemorrhage [14]. Prenatal dexamethasone may reduce virilization in high-risk pregnancies if initiated before nine weeks, but because CAH is autosomal recessive, only one in eight fetuses is likely to benefit, and the therapy remains controversial [15,16]. This treatment should only be considered after individualized counseling. In this case, genetic testing was provided, and follow-up is ongoing with pediatric endocrinology. We recommend avoiding feminization or gender-assignment surgery during childhood, allowing the child to participate in future decisions about their body.

## Conclusions

Fetal ovarian cysts are increasingly detected on prenatal ultrasound, and when accompanied by ambiguous genitalia, CAH should be strongly suspected, especially in settings without universal newborn screening. Prenatal ultrasound enables the early detection of structural abnormalities and can guide timely interventions that improve outcomes. Further studies are needed to determine the optimal management strategy - expectant monitoring versus gonadal-sparing surgery - through multidisciplinary care involving maternal-fetal medicine specialists, neonatologists, fetal and pediatric surgeons, geneticists, reproductive specialists, and endocrinologists.
